# Supplementary material for: ‘Age specific variations in ovarian reserves in healthy fertile and infertile women: A cross sectional study
Source: PLoS One. 2024 Oct 24;19(10):e0308865. doi: 10.1371/journal.pone.0308865 (PMC11500972; doi:10.1371/journal.pone.0308865)
Supplement: Attachment — Submitted filename: RESPONSE TO EDITORS & REVIEWERS.docx [file pone.0308865.s002.docx]

RESPONSE TO EDITORS

1. Formatting done as per Journal protocol
2. Raw Data Attached
3. Abstract included after Title page
4. Corrected Reference list attached

RESPONSE TO REVIEWERS

The authors should change the sentence 'Assessment of AFC has been a simple method of predicting the occurrence of menopause and thus the duration of the reproductive lifespan'. Because we know, based on the work of Kim C. et al. ‘Among women aged 45-49 years, undetectable AMH concentrations were associated with a greater than 60% probability of menopause within 5 years, whereas approximately 1/3 of women with no or just one antral follicle experienced menopause within 5 years. Both low and high concentrations of FSH were associated with greater odds of menopause than intermediate concentrations. Models with multiple markers did not improve the prediction of menopause over that afforded by models with single markers’. (Maturitas 2017 Aug:102:18-25).

ANSWER : editing done ( AMH and FSH also added for predicting menopause ).

- In the infertile population, were couples with infertility due to male factor and tubal factor excluded?

ANSWER : Severe Male factor infertility were excluded but mild male factor were included. Tubal factor infertility were all included.

- The p value acronyms in the figures should always be arranged in the same format

Answer : Changed as per requirements

- Explanations on figures and tables should be clearer

Answer : Captions added.

- Spelling and typographical errors in the article should be corrected

Answer : Corrections done.
